# Multidimensional liquid biopsy in bladder cancer: advances in circulating tumor cells, circulating tumor DNA, exosomes, and metabolomics

**DOI:** 10.1093/oncolo/oyaf409

**Published:** 2026-01-23

**Authors:** Dianjie Zeng, Bojian Liu, Fei Deng, Yinhuai Wang, Jiachen Liu, Zebin Deng

**Affiliations:** Department of Urology, The Second Xiangya Hospital at Central South University, Changsha 410011, Hunan, China; Xiangya School of Medicine, Central South University, Changsha, Hunan 410078, China; Xiangya School of Medicine, Central South University, Changsha, Hunan 410078, China; Xiangya Hospital, Central South University, Changsha, Hunan 410008, China; Department of Urology, The Second Xiangya Hospital at Central South University, Changsha 410011, Hunan, China; Department of Nephrology, The Second Xiangya Hospital at Central South University, Changsha, 410011, China; Department of Urology, The Second Xiangya Hospital at Central South University, Changsha 410011, Hunan, China; Department of Urology, The Second Xiangya Hospital at Central South University, Changsha 410011, Hunan, China; Xiangya Hospital, Central South University, Changsha, Hunan 410008, China; Department of Urology, The Second Xiangya Hospital at Central South University, Changsha 410011, Hunan, China; Department of Nephrology, The Second Xiangya Hospital at Central South University, Changsha, 410011, China

**Keywords:** liquid biopsy, bladder cancer, exosome, lipid metabolism, tumor microenvironments

## Abstract

Bladder cancer (BCa), marked by clinical heterogeneity and late diagnosis, remains a global health challenge. The limitations of conventional diagnostics have spurred the advancement of liquid biopsy approaches, which offer minimally invasive tools for early detection, prognosis, and therapeutic monitoring. This review highlights key components of liquid biopsy in BCa, including circulating tumor cells (CTCs), circulating tumor DNA (ctDNA), exosomes, and metabolomics—especially urinary volatile organic compounds (VOCs). Each modality contributes distinct insights into tumor biology: CTCs and ctDNA provide information on tumor genetics and dynamics; exosomes reflect microenvironmental signaling and lipid metabolism; and urinary VOC profiling enables metabolic characterization and early-stage discrimination. We explore how these dimensions complement each other in tracking disease progression, predicting recurrence, and guiding personalized therapy. Emphasis is placed on recent technological advances, clinical utility, and future integration into practice. This multidimensional perspective underscores the transformative potential of liquid biopsy in improving BCa outcomes.

Implications for Practice Multidimensional liquid biopsy approaches, including circulating tumor cells, circulating tumor DNA, and exosomes, offer promising noninvasive tools for bladder cancer management. These biomarkers enable early detection, prognostic assessment, and treatment monitoring. Circulating tumor DNA and circulating tumor cells provide insights into genetic alterations and tumor dynamics, while exosomes reflect microenvironmental and metabolic regulation, particularly through lipid signaling. Integration of these biomarkers, along with metabolomic profiling (e.g., urinary volatile organic compounds), may enhance diagnostic accuracy and support precision oncology in clinical practice.

## Introduction

Bladder cancer (BCa), a malignancy originating in the tissues of the urinary bladder, is characterized by its heterogeneity in presentation and progression.^1^^,^^2^ In 2018, BCa was classified as the 10th most common cancer globally.^3^ Notably, its incidence is significantly higher in men, where it ranks as the sixth most common cancer.^3^ The global distribution of BCa incidence reveals a higher concentration in developed regions.^4^ According to GLOBOCAN 2020 data,^5^ there were approximately 573 000 new cases and 213 000 deaths, with age-standardized incidence rates notably elevated in Europe and North America. These statistics elucidate the substantial burden of this malignancy, emphasizing the need for improved diagnostic and prognostic strategies.

Numerous risk factors have been identified for BCa, mirroring the multifactorial nature of pathogenesis. Tobacco ­smoking^6^ is a primary risk factor, accounting for approximately 50% of all cases. The risk increases with the intensity and duration of smoking and remains higher in ex-smokers compared to nonsmokers, even decades after cessation.^7^^,^^8^ Occupational exposure to certain chemicals, especially in industries dealing with dyes,^9^ rubber,^10^ leather,^11^ textiles,^12^ and paint,^13^ is another well-established risk factor. Besides, chronic bladder ­inflammation,^14^ often related to urinary tract infections,^15^ ­urinary calculi,^16^ or prolonged use of urinary catheters, also contributes to an increased risk of BCa. Other factors include exposure to radiation therapy,^17^ especially for previous cancers such as prostate or cervical cancer, and the use of certain chemotherapy drugs like cyclophosphamide.^18^

The insidious onset of BCa, characterized by nonspecific or absent initial symptoms, frequently leads to delayed ­diagnosis.^19^ The clinical presentation of BCa, particularly in its early stages, can often be nonspecific or entirely absent, which substantially increases the difficulty of early diagnosis.^20-22^ The most common symptom associated with BCa is hematuria,^23^ which may be either microscopic or macroscopic, commonly observed in urological malignancies, including renal cell carcinoma and urothelial carcinoma.^24^ Additional symptoms may include urinary frequency, urgency, and dysuria, which are not pathognomonic of BCa.^2^ The subtle or nonspecific symptoms of early BCa frequently result in delayed diagnosis and poorer clinical outcomes.^25^^,^^26^ Therefore, a significant proportion of patients present with advanced disease stages or with metastatic spread, complicating treatment and prognosis.

Bladder cancer can be categorized based on invasiveness and extent. Non-muscle-invasive BCa (NMIBC),^27^ which represents the majority of initial diagnoses, requires vigilant surveillance and intravesical therapy due to its high recurrence rate. Muscle-invasive BCa (MIBC),^28^ on the other hand, demands more aggressive treatment approaches. The early detection of MIBC is of utmost importance due to the substantial variations in treatment options and prognosis depending on the stage of diagnosis.^29^

Traditional BCa therapies (surgery, radiation, ­chemotherapy)^30^ face specificity and toxicity limitations. For localized NMIBC, transurethral resection (TURB) with intravesical bacillus Calmette–Guérin (BCG) remains standard.^31^ Muscle-invasive BCa typically requires radical cystectomy with neoadjuvant chemotherapy, which improves survival.^28^^,^^32^ Systemic cisplatin-based chemotherapy remains common for metastatic disease,^33^ though checkpoint inhibitors now offer alternatives, especially for cisplatin-ineligible patients.^34^^,^^35^ Notably, antibody-drug conjugates, exemplified by enfortumab vedotin, combined with pembrolizumab (an anti-PD-1 checkpoint inhibitor), are progressively replacing cisplatin-based chemotherapy as the first-line regimen for metastatic BCa, particularly in patients experiencing recurrence within 12 months after treatment completion.^36^ Despite improvements in surgical techniques and multimodal therapy, 5-year survival rates for patients with MIBC remain suboptimal. Virtually all deaths from BCa result from the muscle-invasive disease that recurs or metastasizes after local therapy.^37^

Early detection of BCa is pivotal for effective management and mortality reduction.^38^^,^^39^ Initial-stage diagnosis, particularly of NMIBC, correlates with a more favorable prognosis and diverse treatment modalities, contrasting with the limited options available in advanced BCa. Current diagnostic methodologies for BCa present considerable limitations. Although invasive cystoscopy remains the diagnostic gold standard, its repetitive use causes considerable patient discomfort. More critically, cystoscopy exhibits limited resolution in detecting flat lesions such as carcinoma in situ (CIS) or early-stage tumors.^40^ Emerging imaging modalities such as CT urography and multi-parametric MRI remain constrained to detecting advanced-stage tumors (T3b/T4), exhibiting markedly reduced sensitivity for early lesions or non-muscle-invasive disease.^41^ The unavailability of reliable, noninvasive screening modalities impedes early BCa identification. The utility of tumor-specific biomarkers in screening is constrained by complexities in establishing their analytical and clinical validity.^42^ Currently used biomarkers, such as nuclear matrix protein (NMP22) and bladder tumor antigen (BTA), appear to correlate with BCa differentiation status. However, in clinical practice, these markers exhibit limited diagnostic performance: NMP22 demonstrates only 50% sensitivity for low-grade tumors,^43^ while BTA achieves 65% sensitivity,^44^ both accompanied by suboptimal specificity. The majority of these biomarkers, available as laboratory-developed tests, lack rigorous clinical validation.

**Figure 1.**
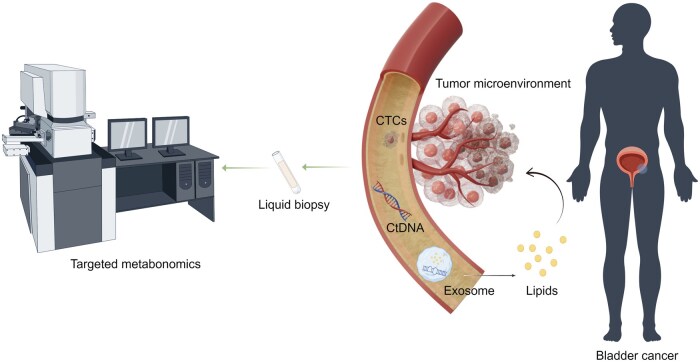
Overview of liquid biopsy in bladder cancer. Liquid biopsy is emerging as a promising method. Liquid biopsy involves the collection and analysis of different tumor components, including circulating tumor cells (CTCs), circulating tumor DNA (ctDNA), and exosomes. Among these, exosomes secreted by primary tumor cells or circulating tumor cells carry messenger RNA (mRNA), micro RNA (miRNA), and a variety of metabolites, which also influence the tumor microenvironment. High-throughput metabolomics analyzes the levels of different lipid metabolites in exosomes by exosome-specific targeted metabolomics.

Fortunately, advances in the field of biomarker research and the advent of novel diagnostic technologies, such as liquid biopsy, herald promising prospects for the early detection of BCa.^45^ Liquid biopsy encompasses a range of analytes—including circulating tumor cells (CTCs), circulating tumor DNA (ctDNA), and exosomes—that enable noninvasive, real-time profiling of tumor biology. In addition, metabolomics, particularly through urinary volatile organic compounds (VOCs), provides complementary metabolic information that may further enhance diagnostic precision. These emerging techniques have the potential to revolutionize BCa diagnostics, enabling earlier therapeutic interventions and consequently improving patient prognoses.^46^ In this comprehensive review, we aim to provide a multidimensional overview of liquid biopsy in BCa. We synthesize recent advances across CTCs, ctDNA, exosomes, and metabolomic profiling, with an emphasis on their biological significance, clinical utility, and potential for integration into precision oncology (Figure 1). By examining the interplay between these biomarkers and tumor behavior—including metastasis, treatment response, and recurrence—we seek to highlight their individual and collective contributions to improving BCa diagnosis, prognosis, and management.

## Liquid biopsy in the diagnosis and prognosis of BCa

Conventional diagnostics like cystoscopy, though clinically useful, are limited by invasiveness and variable accuracy. Cystoscopy shows poor sensitivity for CIS, with a meta-analysis reporting pooled sensitivity of 0.75 (95% confidence interval [CI]: 0.70–0.79) for BCa, but only 0.0075% for CIS and 0.87% for Ta-stage tumors.^47^ This is partly due to CIS lacking exophytic growth and angiogenesis, which reduces optical contrast. Confounding factors such as gross hematuria further reduce specificity, leading to a 40% false-positive rate.^48^ Urinary calculi can also cause false positives due to mucosal inflammation. These challenges worsen when tumor cellularity is low. Current urinary biomarkers for BCa diagnosis,^42^ including NMP22, BTA, and cytology, demonstrate inconsistent accuracy in early detection and limited reliability in distinguishing malignant from benign urological conditions. Specifically, studies have shown that both NMP22 and urine cytology exhibit suboptimal sensitivities of 44.1% and 32.3%, respectively, for early-stage BCa detection.^49^ Similarly, the BTA test achieves only 67% sensitivity for early-stage tumors.^50^ Although these markers are highly specific in some studies, their low sensitivity limits their utility in early detection, and benign conditions such as urinary tract infections or hematuria can still produce false positives due to biological overlap.

Liquid biopsy has emerged as a revolutionary technique in oncology, offering noninvasive cancer monitoring through analysis of CTCs, ctDNA, and exosomes in blood, urine, and other fluids.^51^ By overcoming tissue biopsy heterogeneity, it reveals molecular profiles of BCa pathogenesis. This approach enhances early detection, tracks tumor evolution, and informs therapeutic strategies. Its repeatability and capacity to address tumor heterogeneity mark a paradigm shift in BCa diagnostics and management. The noninvasive nature of this technique, coupled with its capability to address tumor heterogeneity, represents a noteworthy advancement compared to conventional diagnostic methods.

### Circulating tumor cells

Circulating tumor cells^52^ are tumor cells shed from the primary tumor that escape immune surveillance. They are closely linked to early cancer detection, prognosis, monitoring, and assessing the risk of metastasis. By entering the bloodstream, these cells offer insights into the molecular and cellular features of BCa. Analyzing CTCs through genomic and transcriptomic profiling helps reveal tumor biology and reflect the characteristics of the primary tumor. Advanced techniques like single-cell ­sequencing^53^ allow for a deeper understanding of CTCs, aiding in prognosis, tracking metastasis, identifying therapy resistance, and discovering potential targets for treatment.

The seminal work by Naoe et al.^54^ introduced the use of the CellSearch test for evaluating CTCs in BCa patients, establishing their role in metastasis by detecting CTCs in 71.4% of metastatic urothelial carcinoma cases, but not in localized BCa. This finding highlighted the potential of CTCs as markers of metastatic progression. Subsequent studies further supported their prognostic value. Gallagher et al.^55^ found that in patients with untreated or progressive metastatic urothelial carcinoma, the presence of one or more CTCs correlated with a higher incidence of metastasis at various sites. These results support the idea that CTCs may indicate advanced disease. Additionally, research by Rink et al.^56^ confirmed the prognostic significance of CTCs in patients undergoing radical cystectomy for BCa. Their study revealed that the presence of CTCs was significantly associated with worse oncological outcomes, including increased risks of disease recurrence, reduced overall survival, and elevated cancer-specific mortality. Although multivariable adjustment was not performed, these findings suggest that CTCs may reflect an elevated risk of early systemic disease and hold potential as supplementary prognostic indicators beyond conventional clinicopathological factors. The applicability of the CellSearch test also extends to NMIBC. Gazzaniga et al.^57^ have demonstrated that the presence of CTCs in NMIBC patients is correlated with an increased risk of tumor recurrence and progression to muscle-invasive disease. This finding highlights the potential significance of CTCs in identifying patients with clinically under-staged BCa, who may benefit from more aggressive treatment approaches.

However, the clinical application of CTCs remains limited. Although numerous studies have reported associations between CTC presence and adverse outcomes in BCa patients,^58^ these findings are often constrained by small sample sizes and heterogeneous detection methods. Recent efforts have expanded into molecular profiling of CTCs. For instance, HER2 expression has been detected in approximately 14% of non-metastatic BCa patients before radical cystectomy, with high concordance between human epidermal growth factor receptor 2 (HER2)-positive CTCs and primary tumor tissue.^59^ Furthermore, advanced microfluidic platforms—such as epithelial cell adhesion molecule (EpCAM)/epidermal growth factor receptor (EGFR)-enriched graphene oxide chips—have enabled the detection of invasive markers including EGFR, HER2, cluster of differentiation 31 (CD31), and a disintegrin and metalloproteinase 15 (ADAM15), as well as CTC-derived RNA signatures associated with metastasis and treatment resistance.^60^ While these molecular insights highlight the promise of CTCs as liquid biopsy tools for personalized therapy, their clinical utility remains exploratory due to the lack of large-scale validation and standardized workflows. In addition, the low abundance, phenotypic heterogeneity, and temporal fluctuation of CTCs continue to pose technical challenges, with current methods displaying wide variability in sensitivity and specificity.^60^ Integrating high-efficiency isolation strategies with multiplexed molecular profiling may be essential to fully realize the diagnostic and prognostic value of CTCs in BCa.

### Circulating tumor DNA

Circulating tumor DNA (ctDNA),^61^ comprising fragmented DNA shed by tumor cells into the bloodstream, serves as a reservoir of tumor-specific genetic information, which also harbors mutations and other distinctive markers reflective of the originating neoplasm. DNA sequencing of ctDNA from liquid biopsy samples enables the detection of a spectrum of genetic alterations, including insertions and deletions (Indels), fusion genes, single nucleotide variants (SNVs), and copy number variations (CNVs).

Recent advances in urinary ctDNA detection enhance liquid biopsy’s role in early cancer screening. Aberrant methylation patterns, detectable via methylated DNA immunoprecipitation and sequencing, improve sensitivity for early tumors.^62^ Concurrently, sequencing innovations like Van Der Pol’s ONT framework enable rapid genomic/fragmentomic Cell-Free DNA (CfDNA) analysis, identifying tumor-derived long ctDNA in urine.^63^ In targeted detection, Nikkola et al. employed the UroScout multi-gene panel for deep sequencing of urinary sediment DNA, enabling high-precision tumor diagnosis^64^; meanwhile, pre-analytical optimizations (e.g., Ward’s AMPure XP bead-based selection) allow low-input serum assays without tumor tissue.^65^ These breakthroughs establish multidimensional ctDNA analysis for minimally invasive cancer management.

In the field of BCa diagnostics, ctDNA analysis presents notable advantages compared to other liquid biopsy components, specifically CTCs.^66^ CtDNA is typically found in higher concentrations in peripheral blood, making it more easily detectable and analyzable.^67^ The extensive genomic profiling offered by ctDNA analysis provides valuable information on tumor heterogeneity, encompassing both spatial and temporal aspects. Additionally, it allows for the identification of somatic mutations that may not be present in the corresponding tumor tissues,^68^ thereby offering a more comprehensive genomic understanding of the malignancy.

Previous studies have highlighted the prognostic role of various ctDNA mutations in BCa (Table 1), particularly post-cystectomy. In a prospective study^77^ of 50 MIBC patients undergoing neoadjuvant cisplatin-gemcitabine (Cis-Gem) followed by cystectomy, ctDNA detected in plasma after cystectomy was associated with systemic recurrence, with a median difference of 101 days between ctDNA detection and clinical diagnosis of recurrence. Besides, Carrasco et al.^71^ found that ctDNA status at cystectomy was associated with a higher pathological stage of BCa progression.

**Table 1.** Circulating tumor DNA (CtDNA) as potential biomarkers for bladder cancer.

Authors (Ref.)	*N*	Method	Mutations	Application	Effect	Clinical context
**Shohdy et al.** ^69^	182	NGS, WES	TSC1 E1044fs HRAS G12S	Predict disease progression	OS (*P* = 0.03)	Advanced
**Ravi et al.** ^70^	45	NGS	TP53 TERT	Treatment monitoring	ctDNA alterations correlate with ICI resistance	Advanced, 39 patients receiving ICI and 6 receiving platinum-based chemotherapy
**Carrasco** ^71^	37	Quant-iT PicoGreen dsDNA kit and ddPCR	TERT	Prognostic biomarker	cfDNA, ctDNA at 4 months post-RC predict progression (HR 5.290; *P* = 0.033) and survival (HR 4.199; *P* = 0.038)	MIBC patients after RC
**Szabados** ^72^	92	WES of tumor tissue and multiplex PCR-NGS ctDNA assay	CCL4 MMP9	Prognostic biomarker	ctDNA status prognostic at all time points	Patients received atezolizumab, part of patients did not undergo cystectomy
**Vandekerkhove et al. (2021)** ^67^	104	WES, QIAGEN DNeasy Blood and Tissue Kit	APOBEC	Predict prognosis	OS (*P* = 0.01), PFS (*P* = 0.02)	At least one distant metastatic lesion (M1)
**Powles et al.** ^73^	581	WES, multiplex PCR	KRT6C KNL1	Predict recurrence/predict treatment response	DFS (*P* < 0.0001); ctDNA for MRD predicts immunotherapy response	Undergo surgery and adjuvant atezolizumab versus observation
**Zhang et al.** ^74^	82	Targeted sequencing	FGFR3 PIK3CA	Predict prognosis	DFS (*P* = 0.0146)	NMIBC patients receiving TUR of bladder
**Christensen et al.** ^38^	68	WES, ultra-deep sequencing	ERCC2	Predict metastatic recurrence/monitoring of therapeutic efficacy	Sensitivity 100%, specificity 98%; ctDNA changes correlate with recurrence (*P* = 0.023)	Advanced, before and after cystectomy and during chemotherapy
**Grivas et al.** ^75^	124	Exon sequencing	BRCA1 RAF1	Predict prognosis	OS (*P* = 0.07), FFS (*P* = 0.016)	Patients received prior therapy with platinum, 21 with a taxane, and 10 with a PD-1/PD-L1 inhibitor, respectively
**Sundahl et al.** ^6^	9	RT-PCR	TP53 TERT	Response monitoring	Predicts treatment response before imaging	Pembrolizumab combined with radiotherapy in metastatic BCa
**Birkenkamp-Demtröder et al.** ^77^	60	WES, ddPCR	PIK3CA FGFR3	Monitoring recurrence	Detected earlier recurrence vs. imaging	Metastatic relapse
**Raja et al.** ^78^	29	Targeted sequencing	TP53 ARID1A	Predict treatment response	Early ctDNA changes identify checkpoint inhibitor non-responders	Accept durvalumab, an anti-PD-L1 therapy
**Vandekerkhove et al.** ^79^	51	Targeted and exome sequencing	TP53 RB1 MDM2	Revealing aggressive mutations in metastatic BCa	Detected aggressive mutations in 95% of metastatic BCa	51 patients with aggressive BCa, including 37 with metastatic disease
**Patel et al.** ^80^	17	TAm-Seq, WGS	TP53	Monitoring recurrence	PPV 100%, NPV 85.7% for recurrence	MIBC accept neo-adjuvant chemotherapy
**Khagi et al.** ^81^	69	NGS	APOBEC	Predict treatment response	ctDNA hypermutation predicts improved response, PFS, OS	Patients received checkpoint inhibitor-based immunotherapy
**Birkenkamp-Demtröder et al.** ^82^	12	NGS, ddPCR	FGFR3 TERT PIK3CA	Predicts disease progression and residual disease	Predicted progression (*P* = 0.032)	Recurrent or progressive/metastatic NMIBC
**Hauser et al. (2013)** ^83^	227	Methylation-specific PCR	TIMP3 APC RARB	Discrimination of patients with BCa from healthy individuals	Sensitivity 62%, specificity 89%	75 patients NMIBC, 20 MIBC, 48 TURB without BCa, 31 benign disease, 53 healthy individuals
**Lin et al.** ^84^	168	Methylation-specific PCR	CDH13	Diagnostic biomarker	Detected in 30.7% of patients, higher in advanced BCa	Metastatic BCa
**Ellinger et al.** ^85^	45	Restriction endonuclease-based assay, qRT-PCR	APC DAPK GSTP1	Increase the accuracy of the diagnosis of BCa	Sensitivity 80%, specificity 93%	Patients with BCa undergoing cystectomy
**Valenzuela et al.** ^86^	135	Methylation-specific PCR	p53 p16INK4a	Diagnostic biomarker	AUC 95%, sensitivity 22.6%, specificity 98%	BCa patients
**Domínguez et al.** ^87^	27	5–4520 kit, QIAamp Blood kit, PCR	p16(INK4a)	Diagnostic biomarker	Detected in 40% of patients	BCa patients

Abbreviations: AUC, area under the curve; BCa, bladder cancer; ctDNA, circulating tumor DNA; ddPCR, droplet digital PCR; DFS, disease-free survival; dsDNA, double-stranded DNA; FFS, failure-free survival; ICI, immune checkpoint inhibitor; MIBC, muscle-invasive BCa; NGS, next-generation sequencing; NMIBC, non-muscle-invasive BCa; NPV, negative predictive value; WES, whole exome sequencing; OS, overall survival; PCR, polymerase chain reaction; PD-1/PD-L1, Programmed Death-1/Programmed Death-Ligand 1; PFS, progression-free survival; PPV, positive predictive value; qRT-PCR, quantitative reverse transcription PCR; RC, rectal cancer; RT-PCR, reverse transcription-polymerase chain reaction; TUR(B), transurethral resection of a bladder tumor; WGS, whole genome sequencing.

Recent large clinical trials have shown the prognostic value of ctDNA detection in BCa. The Atezolizumab in Bladder Cancer (ABACUS) phase 2 trial^72^ evaluated atezolizumab as neoadjuvant therapy in MIBC patients and found that ctDNA levels changed before and after treatment. Posttreatment ctDNA positivity was linked to higher recurrence, lymph node involvement, and advanced T stage. Similarly, the A Study of Atezolizumab as Adjuvant Therapy in Muscle-Invasive Bladder Cancer (IMVIGOR010) phase 3 trial^88^ showed that patients with detectable ctDNA after cystectomy had a higher recurrence risk without adjuvant therapy, while those receiving atezolizumab had better disease-free and overall survival. The Neoadjuvant Ipilimumab plus Nivolumab in Muscle-Invasive Bladder Cancer (NABUCCO) trial,^89^ which tested different doses of ipilimumab and nivolumab in advanced MIBC, found that undetectable ctDNA was associated with complete pathological response and longer progression-free survival. These findings support the role of ctDNA as a promising biomarker for monitoring treatment response and recurrence risk in the neoadjuvant setting.

The potential of ctDNA in BCa extends to early disease detection, monitoring of drug resistance, companion diagnostics, and prognostic assessments. However, ctDNA levels and genetic profiles can vary significantly among patients with similar tumor types and stages, presenting challenges in standardizing its clinical application. Additionally, the accumulation of somatic mutations in nonmalignant lesions, particularly in the context of aging, may confound the interpretation of oncogenic mutations detected in ctDNA^90^ BCa. Consequently, ctDNA analysis is most effectively utilized within the structured framework of clinical trials. Recent recommendations by major oncological societies, such as the European Society of Medical Oncology (ESMO),^91^ endorse ctDNA testing for genotyping and treatment selection in advanced cancer patients. However, caution is advised in its application for cancer screening, molecular residual disease assessment, molecular relapse monitoring, and early treatment response evaluation. The dynamic nature of ctDNA in oncology necessitates a balanced view of its opportunities and limitations in clinical practice.

## Exosomes

Exosomes,^92^ which are nanoscale extracellular vesicles with a diameter typically ranging from 40–160 nm, were initially discovered in reticulocytes and have subsequently gained recognition for their substantial involvement in cancer biology, particularly in relation to BCa. These vesicles are distinguished by their capacity to transport diverse cellular constituents, including nucleic acids, proteins, lipids, and metabolites, thereby mirroring the cellular and molecular attributes of their originating cells.^92^ The presence of exosomes in different bodily fluids, such as blood and urine, suggests their potential utility as noninvasive biomarkers for cancer diagnostics. Urine-derived exosomes, which reside in the liquid microenvironment of BCa, have emerged as promising diagnostic biomarkers.^93^

Extensive research^94^ has been conducted to investigate the exosomal cargoes found in BCa (Table 2). The composition of exosomes, which carry micro ribonucleic acid (miRNAs) and other molecules, offers valuable insights into the biological behavior of BCa and potential pathways for metastasis and progression. For instance, Lung Cancer Associated Transcript 1 (LUCAT1)-containing exosomes enhances the stemness phenotype and chemoresistance of BCa cells by upregulating high mobility group AT-hook 1 (HMGA1) expression, thereby promoting oncogenicity.^106^ Exosome miR-184 helps BCa escape immunity by acting on AKR1C3.^107^ Furthermore, BCa downregulates exosomal miR-152-3p levels to promote angiogenesis.^108^ Studies demonstrate significant alterations in exosomal expression profiles of BCa patients. Analyzing the content of exosomes, including specific miRNAs, holds promise for the identification and treatment of BCa at an early stage.^109^ Unlike ctDNA and CTCs, exosomes provide a more comprehensive overview of heterogeneity and evolution, rendering them particularly valuable in this context.

**Table 2.** Exosomes as potential biomarkers for bladder cancer.

Authors (Ref)	Biomarker	*N*	Methods	test environment	Application	Effect
**Yang et al.** ^95^	circTRPS1	90	ExoQuick Exosome Precipitation Solution	Urinary-derived	Predict prognosis	OS (*P* = .01)
**Yin et al.** ^96^	miR-663b	122	ExoQuick‐TC Exosome Precipitation Solution	Plasma-derived	Predict disease progression	Disease progression (*P* < .05)
**Yan et al.** ^97^	LINC00355	57	miRNA microarray	Plasma-derived	Predict disease progression	Disease progression (*P* < .05)
**Cai et al.** ^98^	miR-133b	11	Total exosome isolation reagent	Plasma-derived	Predict disease progression	Disease progression (*P* < .05)
**Sabo et al.** ^99^	miR-126-3p	93	Deep sequencing	Plasma-derived	Predict prognosis	OS (*P* < .05)
**Elsharkawi et al.** ^100^	Tumor-derived exosomes	82	ExoQuantTM overall exosome capture and quantification assay kit, ELISA	Urinary and Plasma-derived	Diagnostic biomarker	Sensitivity = 82.4%, specificity = 100%
**Zhang et al.** ^101^	LINC00355	520	ExoQuick exosome purification kit	Plasma-derived	Predict prognosis	RFS (*P* = .01)
**Wang et al.** ^102^	H19	104	ExoQuick exosome purification kit	Plasma-derived	Diagnostic biomarker	AUC = 85.1%, sensitivity = 74.07%, specificity = 78.8%
**Zheng et al.** ^103^	lncRNA-PTENP1	110	ExoQuick exosome purification kit	Plasma-derived	Diagnostic biomarker, predict disease progression	AUC = 74.3%, sensitivity = 65.4%, specificity = 84.2%, predict disease progression (*P* < .05)
**Chen et al.** ^104^	circRNA-PRMT5	119	Electron microscopy	Urinary and Plasma-derived	Predict prognosis	OS (*P* = .028)
**Xue et al.** ^105^	lncRNA-UCA1	60	ExoQuick exosome purification kit	Plasma-derived	Diagnostic biomarker	AUC = 87.83%, sensitivity = 80%, specificity = 83.33%

Abbreviations: AUC, area under the curve; OS, overall survival.

Recent studies highlight the importance of exosomes from BCa cells or biofluids in understanding disease progression. Exosomal miR-375 and miR-146a have been proposed as urinary biomarkers for high- and low-grade BCa, respectively.^110^ Furthermore, the identification of exosomal contents such as HOX transcript antisense intergenic RNA (HOTAIR) and additional long non-coding RNA **(**lncRNAs in BCa) underscores their potential as diagnostic and therapeutic targets.^111^ Exosomes are being explored for treatment purposes as well. Engineered exosomes can deliver antitumor drugs or genetic material. For instance, Chen et al. found that BCa cell-derived exosomal exosomal circular RNA TRPS1 (circTRPS1) can regulate reactive oxygen species and promote CD8+ T cell exhaustion via the circTRPS1/miR-141-3p/GLS1 axis.^111^ This use of exosomes leverages their natural ability to carry molecular cargo, suggesting a novel therapeutic strategy for BCa.

Emerging proteomic analyses of urinary exosomes have identified distinct protein signatures with diagnostic potential in BCa. Notably, levels of α1-antitrypsin, apolipoprotein E, and BTA are significantly higher in the urine of BCa patients than in healthy controls.^112^ Histone H2B1K (m/z 5947 peak), present in normal exosomes, is overexpressed in BCa and validated by matrix-assisted laser desorption/ionization time-of-flight (MALDI-TOF) mass spectrometry.^113^ Additionally, proteins such as MMP12, MMP7, and heme oxygenase-1 show BCa-specific expression compared to both healthy individuals and prostate cancer patients, emphasizing the distinct molecular profile of BCa-derived exosomes.^114^ These findings support the growing potential of exosome-based protein biomarkers for BCa detection.

Despite significant progress in exosome research in BCa, challenges remain in standardizing exosome isolation and analysis methodologies. Future research should focus on identifying reliable exosomal biomarkers, elucidating their role in BCa biology, and developing standardized methods for their clinical application. As research advances, exosomes might emerge as crucial elements in BCa diagnosis, prognosis, and personalized treatment.

## Lipid metabolic targets of exosomes in BCa

### Background

The treatment of BCa, particularly at advanced stages, remains a significant challenge, often marked by metastasis and resistance to conventional therapies. Emerging evidence suggests that ­exosomes,^115^^,^^116^ small extracellular vesicles, play a critical role in the progression and metastasis of BCa. These exosomes carry diverse bioactive molecules, including lipids, which can influence cancer cell behavior and the tumor microenvironment.

Exosomes have been identified as key players in lipid metabolic reprogramming,^117-119^ in the complex milieu of BCa, which play a pivotal role in mediating lipid metabolic processes, crucial for tumorigenesis and progression. These exosomes carry various lipid species that can alter the lipid composition of recipient cells, thereby influencing tumor growth, metastasis, and treatment response. The lipid contents of exosomes derived from BCa cells exhibit distinct profiles, which are reflective of the metabolic state of the cancer cells and the tumor microenvironment. The unique lipid composition of BCa-derived exosomes presents novel opportunities for targeted therapy.^120^ Manipulating exosomal lipid content may disrupt the metabolic support they provide to tumor cells. Additionally, exosomes can deliver lipid-based therapeutics directly to tumors, improving drug efficacy and minimizing systemic side effects.

Lipidomic analysis of exosomes in liquid biopsies offers a promising noninvasive approach for BCa diagnosis and prognosis. Alterations in exosomal lipid composition may serve as biomarkers for early detection, disease monitoring, and treatment response prediction.^121^ Moreover, the lipid profiles of exosomes could potentially be used to stratify patients for personalized therapeutic interventions. Therefore, future research should focus on elucidating the specific roles of different lipid species in BCa exosomes, their interaction with cancer cells, and the tumor microenvironment.^122^ Additionally, the development of advanced lipidomic techniques will be crucial for the detailed analysis of exosomal lipid profiles, enabling the identification of novel biomarkers and therapeutic targets.

### Mutually regulation: exosomes and lipid metabolism in BCa

Lipid metabolism assumes a crucial function in the synthesis and discharge of exosomes, as well as their engagement with target cells, which can be ascribed to the indispensable role of lipids as constituent elements within exosomes.^118^ The lipid bilayer of exosomes encompasses diverse lipid constituents, such as sphingomyelin, cholesterol, and ceramides, which exert influence over cargo assortment, exosome secretion, structure, and signal transduction mechanisms. The cellular lipid metabolism state (cholesterol synthesis, fatty acid oxidation) directly modulates the lipid composition of exosomes.

Lipids play a key role in exosome biogenesis by shaping membrane structure and regulating secretion. Sphingolipid- and cholesterol-rich lipid rafts act as platforms for Endosomal Sorting Complex Required for Transport components (e.g., Alix/TSG101),^123^ which are essential for exosome maturation. Sphingomyelin deficiency disrupts CD63 distribution and impairs exosome formation,^124^ while ceramide promotes membrane budding by inducing curvature through its conical shape. This process is regulated by acid sphingomyelinase (ASM), which generates ceramide and enhances exosome secretion.^125^ Cholesterol and ceramide together form a regulatory network that drives exosome biogenesis.

Conversely, exosomes regulate lipid metabolism bidirectionally by delivering functional molecules. Epigenetically, exosomal miR-33a inhibits ATP-binding cassette transporter A1 (ABCA1),^126^ blocking cholesterol efflux and causing intracellular accumulation, particularly under microenvironmental stress. Concurrently, they transfer intact lipid-metabolizing enzymes like lipoprotein lipase,^127^ enhancing triglyceride hydrolysis. While miRNA-mediated chronic regulation promotes carcinogenesis, enzyme delivery enables acute metabolic adaptation. These complementary mechanisms maintain exosome-driven lipid homeostasis.

Exosomes critically regulate lipid metabolism in BCa by transferring lipids and biomolecules between cells.^94^ They promote cancer cell proliferation, invasion, and metastasis while reshaping the tumor microenvironment through lipid-mediated signaling. Clinically, their lipid bilayer stability protects cargo from degradation, making them ideal noninvasive biomarkers for BCa diagnosis and tumor staging. Therapeutically, engineered exosomes (e.g., Exo-miR-138-5p,^94^ MSC-derived miR-139-5p^128^) effectively penetrate tumors to suppress growth. Therefore, exosomes are central to the lipid metabolic processes in BCa, influencing both the local and systemic disease progression, which not only contribute to our understanding of BCa pathogenesis but also hold promise in refining diagnostic and prognostic strategies through their unique lipid metabolic roles.

The complex interplay between lipid metabolism and exosome biology presents novel opportunities for comprehending the advancement and management of cancer. Specifically, modifications in lipid metabolism have the potential to impact exosome-mediated intercellular communication within the tumor microenvironment, thereby exerting influence over cancer cell invasion, metastasis, and resistance to therapeutic agents. Consequently, forthcoming investigations ought to prioritize the examination of distinct lipid constituents in the process of exosome formation, as well as the targeting of these pathways for therapeutic interventions in the context of cancer.

### Metabolomics of BCa

There has been a growing interest in investigating urinary VOCs as potential biomarkers for the detection and staging of BCa.^129-131^ The utilization of gas chromatography-mass spectrometry (GC-MS)-based metabolomics and electronic-nose (e-nose) sensors has been instrumental in these investigations.^102^ Metabolomics, a pivotal component of this undertaking, entails the examination of low molecular weight metabolites generated via cellular mechanisms.^132^ These metabolites, generally spanning from 50 to 200 Da, are present in various biological matrices, such as urine. They function as indicators of physiological or pathological conditions, thus conferring significant worth in cancer research for the noninvasive identification of diagnostic biomarkers.

Urine, by virtue of its close proximity to the bladder, presents a highly suitable substrate for investigating BCa. It encompasses approximately 300 VOCs^133^ derived from diverse chemical classes, including aldehydes, ketones, and hydrocarbons, originating from both endogenous metabolic processes and exogenous sources such as diet and the environment. Gas chromatography-mass spectrometry emerges as a preeminent analytical technique for VOC identification.^134^ Its capacity to effectively separate VOCs with exceptional sensitivity and precision, in conjunction with comprehensive mass spectral databases, facilitates meticulous analysis. Techniques such as solid phase microextraction and dynamic headspace methods are employed for volatile extraction, enhancing the efficacy of this approach.^135^ In contrast, e-nose sensors, designed to mimic the human olfactory system, have shown promise in differentiating the odor profiles of urine from cancer patients and healthy individuals. These sensors, which can be electrochemical, resistive, or piezoelectric, offer quick, noninvasive, and cost-effective analysis, making them suitable for clinical applications.

Several studies using GC-MS-based metabolomics have investigated urinary biomarkers of BCa,^136^^,^^137^ which have compared VOC profiles of BCa patients with cancer-free controls or individuals with other urological diseases. The results varied, with some studies showing significant diagnostic performance. For example, sensitivities ranged from approximately 27% to 97%,^138^ specificities from 43% to 94%,^139^ and accuracies from 80% to 89%.^140^ Collectively, identifying BCa-specific biomarkers and implementing sensitive detection platforms (GC-TOF-MS) alongside multi-omics validation can enhance diagnostic sensitivities, specificities, and accuracies. Conversely, pathological heterogeneity (overlapping inflammatory conditions) and limited sample cohorts may compromise these parameters. The capability to discern specific stages or grades of BCa was also explored, demonstrating that different stages of BCa (from Ta/Tis to T4)^140^ could be successfully differentiated from controls. Thus, metabonomics, primarily through the analysis of urinary VOCs using GC-MS and e-nose sensors, offers a promising avenue for the early detection and staging of BCa. The ability to profile these volatile compounds provides critical insights into the disease, aiding in the development of noninvasive diagnostic tools and potentially improving patient outcomes.

## Conclusions and future perspectives

The diagnosis and management of BCa present considerable difficulties due to its diverse nature and frequently subtle onset. The worldwide impact of this malignancy, particularly its elevated occurrence and fatality rates, emphasizes the pressing need for novel diagnostic and therapeutic approaches. Liquid biopsy techniques, such as the examination of CTCs, ctDNA, and exosomes, have recently made significant progress in the field of BCa diagnostics. These innovative methods provide a noninvasive, dynamic, and reproducible means of detecting and monitoring cancer, surpassing the constraints of conventional diagnostic approaches. Notably, the significance of exosomes in BCa has been underscored due to their potential as biomarkers and targets for therapeutic interventions. The regulation of lipid metabolism plays a significant role in governing the formation, structure, and functionality of exosomes within the extracellular milieu. Consequently, a multifaceted interdependence exists between exosomes and lipids. Based on this, metabolomics, particularly through urinary VOCs, has shown promise in early BCa detection. Advanced analytical techniques like GC-MS and electronic-nose (e-nose) sensors have been instrumental in identifying specific VOC profiles associated with BCa stages and grades. In total, the prospective impact of liquid biopsy and metabolomics in early detection, disease progression monitoring, and personalized treatment strategies could potentially revolutionize the management of BCa, ultimately leading to enhanced patient outcomes. Notwithstanding these advancements, there persist challenges, specifically in the standardization of methodologies for liquid biopsy and metabolomics. Subsequent research ought to concentrate on enhancing these techniques, substantiating biomarkers, and incorporating them into clinical practice.

## Data Availability

No new data were generated or analyzed in support of this research.
